# Exercise professional education, qualifications, and certifications: a content analysis of job postings in the United States

**DOI:** 10.3389/fspor.2024.1338658

**Published:** 2024-01-18

**Authors:** Rachele Pojednic, Devin P. O’Neill, Molly G. Flanagan, Alexis Bartlett, Byron LaGary Carter, Mary A. Kennedy

**Affiliations:** ^1^ Department of Health and Human Performance, Norwich University, Northfield, VT, United States; ^2^ Nutrition and Health Innovation Research Institute, School of Medical and Health Sciences, Edith Cowan University, Joondalup, WA, Australia; ^3^ Institute of Lifestyle Medicine, Harvard Medical School, Boston, MA, United States; ^4^ College of Health & Human Services, Troy University, Troy, AL, United States

**Keywords:** exercise, exercise science education, exercise professionals, exercise physiologist, curriculum

## Abstract

**Introduction:**

Growth in the field of clinical exercise science and the potential impacts on overall health and wellbeing have driven the need for qualified, clinically trained, exercise professionals. And yet, it is not well understood what specific credentials employers are seeking when hiring exercise professionals.

**Purpose:**

The purpose of the study was to examine the qualification requirements for professionals seeking employment in exercise science, exercise physiology, kinesiology or equivalent fields.

**Methods:**

Search platforms Indeed.com and USAJobs.gov were examined within a two week period in 2022. Search terms included “Exercise Physiology”, “Exercise Science”, “Exercise Professional”, “Exercise Prescription”, “Exercise Specialist”, and “Kinesiology”.

**Results:**

A total of *n* = 739 jobs were retrieved and *n* = 615 jobs were included: Exercise Science (*n* = 227), Kinesiology (*n* = 210), Exercise Physiology (*n* = 91), Exercise specialist (*n* = 53), and Exercise prescription (*n* = 32). Over 70% of the jobs analyzed required a bachelor's degree with the remainder requiring various levels of education. The primary certification required was personal trainer (*n* = 94), followed by strength and conditioning specialist (*n* = 33), clinical exercise physiologist (*n* = 26), group exercise (*n* = 17), exercise specialist (*n* = 10), and exercise physiologist (*n* = 5). Four job focus areas were determined: academic teaching and research, general fitness and worksite wellness, athletic performance and rehabilitation, clinical exercise specialist all with varying levels of degree and certification requirements.

**Discussion:**

Job postings related to exercise related professions are varied across the United States with wide-ranging education, credentialing and certification requirements. These findings indicate the timely need for outreach to employers to highlight changing credentialing requirements due to evolving accreditation standards.

## Introduction

The field of exercise science has witnessed significant growth and transformation in recent years, driven by advancements in clinical research linking physical activity and disease, increasing awareness of the importance of physical fitness across the lifespan, and the rising demand for professionals specializing in exercise-related interventions. In addition to traditional roles for exercise professionals in fitness and physical activity interventions for general health and athletic performance, a growing clinical demand, both in the US and abroad, has created diverse employment opportunities and advanced career paths for aspiring exercise professionals (1–3). Indeed, the US Bureau of Labor Statistics (BLS) employment snapshot indicates that there are over 21,000 exercise physiologist jobs with a 9% estimated growth rate, or 1,700 new openings for exercise physiologists each year, by 2031 (4).

In order to respond to the growing demand for qualified exercise professionals both in and out of the clinical space (1–5), accreditation standards for degree granting institutions have been established by the Committee on Accreditation for the Exercise Sciences (CoAES) (6) towards potential program accreditation by the Commission on Accreditation of Allied Health Education Programs (CAAHEP) (7) as well as the Council on Accreditation of Strength and Conditioning Education (CASCE) (8). These standards serve as fundamental guidelines for exercise science programs to ensure quality and consistency of education in the field. Specifically, they identify core competencies and learning outcomes that establish standards & guidelines for academic programs (6) and encompass knowledge in areas such as exercise physiology, human anatomy, exercise prescription, and research methodologies. The goal is to enable all graduates with a bachelor's degree in exercise science to effectively assess, design, and implement exercise programs tailored to individual needs. The accreditation standards set by CoAES, CAAHEP, and CASCE have played a crucial role in shaping the job market for exercise physiologists to help ensure graduates from accredited programs possess the necessary skills and knowledge to meet the demands of the evolving field, as well as provide required credentialing to sit for specific certification exams (i.e., American College of Sports Medicine Certified Exercise Physiologist (ACSM-CEP) (9) and the National Strength and Conditioning Association Certified Strength and Conditioning Specialist (CSCS) (10). And yet, despite a highly structured curriculum and certification pipeline, as well as detailed technical requirements required to be recognized as qualified health professionals (1, 5, 11, 12), it is not well understood what specific credentials *employers* are seeking when hiring exercise physiologists.

Approximately a decade ago, a six-month survey was conducted on job search platforms that identified employment opportunities and the education/certification requirements for exercise physiologists or exercise specialists in clinical settings (13). Due to the evolving educational landscape and the dynamic job market, there is a timely need to update the expectations between academic curricula, certification and degree programs, and employment opportunities to ensure university curricula are designed to best prepare students for future careers. The purpose of the current study was to expand upon prior findings (13) to understand current career opportunities and credentialing expectations by employers, both within clinical settings and beyond, for aspiring professionals in exercise science, exercise physiology, kinesiology or equivalent fields.

## Methods

The databases *Indeed.com* and *USAJobs.gov* were searched within a two-week data collection period beginning on March 29, 2022. While other search engines were initially screened (i.e Linked In, ZipRecruiter, ACSM Job Board, and Higher Education Recruitment Consortium) it was determined that there was tremendous redundancy between postings and the two most robust and non-overlapping were chosen (by RP, DO, and MK). Each search engine aggregates jobs from organization websites and job boards. The following search terms were used for both databases: “Exercise Science”, “Exercise Physiology”, “Exercise Specialist”, “Exercise Prescription”, “Exercise Professional”, and “Kinesiology”. Jobs were included if they were in United States, Washington DC and Puerto Rico. Jobs were excluded if they were not directly relevant and related to practicing within the exercise profession (i.e., administrative assistant, security specialist, or IT specialist).

Data from searches were completed and transcribed by four authors (RP, ED, MF and AB). Duplicate job postings were eliminated. Information that was collected and coded included: job title, facility type and location, degree and/or certification requirements, and salary. If multiple degrees or certifications were mentioned in one job posting, each one was counted separately. Thus, it is possible to have totals that are more than the number of postings. Disagreements about inclusion were resolved through a discussion among the four authors involved in the searches to reach consensus. Upon completion of data abstraction two authors (RP and MK) further categorized and profiled job postings based upon the text of the job listing.

## Results

### Descriptive statistics

A total of *n* = 737 jobs were retrieved and screened. One hundred twenty-two (*n* = 122) jobs were excluded because they did not meet relevant careers within the exercise science or health related disciplines. Six hundred and fifteen (*n* = 615) jobs were included in further analysis. A total of *n* = 227 jobs were titled Exercise Science, followed by Kinesiology (*n* = 210), Exercise Physiology (*n* = 91), Exercise specialist *(n* = 53), and Exercise prescription (*n* = 32). Two (*n* = 2) jobs were listed under both “Exercise Physiology” and “Exercise Science”. The search term Exercise Professional did not return any relevant jobs. Over 70% of the jobs analyzed required a bachelor's degree with the remainder requiring various levels of education (Table 1).

**Table 1:** Educational requirements for all job postings for exercise professionals (*n* = 615).

Educational Requirement	Total Job Postings
Bachelor's Degree	437
High School Diploma	59
Masters Degree	35
Associates Degree	31
No Education Noted	27
Doctoral Degree	13
Bachelors or Masters Degree	10
Associates or Bachelors Degree	3

Certification requirements between the job criteria varied greatly. The primary certification requirement was Cardiopulmonary Resuscitation (CPR) and Basic/Advanced Life Support (*n* = 364). For specific certifications related to exercise knowledge, the primary certification required was certified personal trainer (*n* = 94), followed by certified strength and conditioning specialist (*n* = 33), clinical exercise physiologist (*n* = 26), group exercise (*n* = 17), exercise specialist (*n* = 10), and exercise physiologist (*n* = 5). Thirteen (*n* = 13) postings required a more generic “fitness certification”. Ten (*n* = 10) postings required an EKG certification. The major certification issuers mentioned as “preferred” included American College of Sports Medicine (ACSM, *n* = 59), National Strength and Conditioning Association (NSCA, *n* = 38), Aerobics and Fitness Association of America (AFAA, *n* = 31), American Council on Exercise (ACE, *n* = 27), National Academy of Sport Medicine (NASM, *n* = 27), and National Exercise Trainers Association (NETA, *n* = 14). Due to the small total counts, all other certifications and requirements were considered “other” (*n* = 111).

### Job focus area analysis

**Figure 1:**
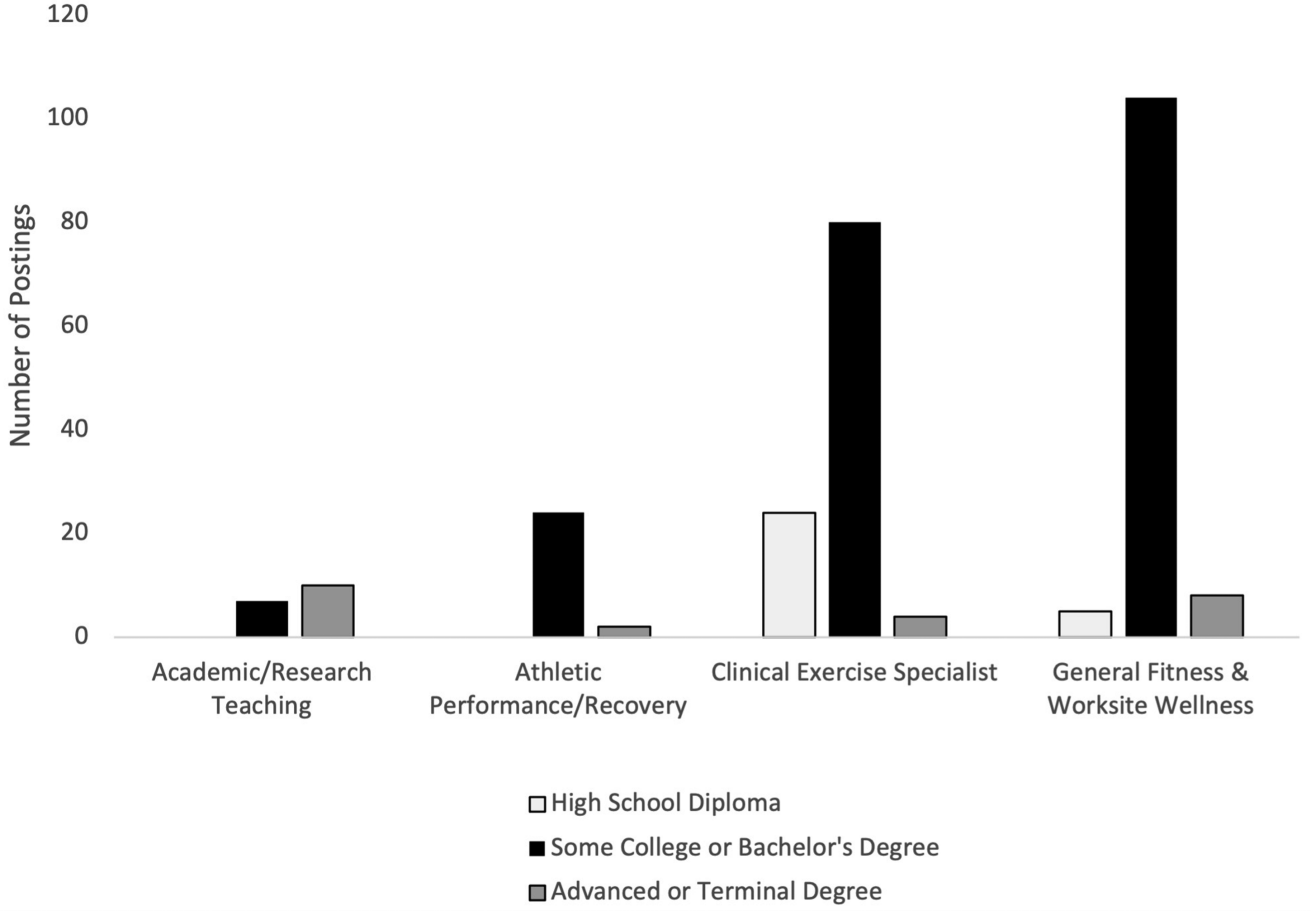
Degree requirements per focus area for exercise professionals.

In a random subset of the larger dataset (*n* = 277) four main job focus areas were categorized and profiled from the job description: academic teaching and research, general fitness and worksite wellness, athletic performance and rehabilitation, clinical exercise specialist. Each is described in this section and outlined in Figure 1.

#### Academic teaching and research

Academic teaching and research positions (*n* = 17) were focused primarily on leading classroom education at the undergraduate level or conducting exercise focused human studies research at academic institutions. Employers were mostly seeking those with an advanced (masters) or terminal (doctoral) degree (59%) while the remainder required a bachelor's degree. Job postings that required an advanced or terminal degree were primarily interested in candidates able to teach at the university level and/or conduct research while those with a bachelor's degree were primarily seeking research program managers or research assistants. Top skills required included: faculty oversight, teaching, curriculum development, data collection and analysis and project management. Examples of key responsibility areas included:

*“Teaching three, one-credit hour labs in the exercise science major coursework; prepare course materials and lessons”*

*“Provides quality education in order to prepare students for their chosen career or post-graduate studies”.*

*“Oversee and manage the recruitment of research participants, schedule research study visits, assist with data and provide oversight with monitoring participant compliance”*

#### General fitness and worksite wellness

General fitness and worksite wellness employees (*n* = 117) were focused on improving exercise behaviors at fitness clubs, private and public work places and in the community at large. Positions included personal trainers, health coaches, group fitness coaches, and wellness program coordinators. The vast majority of positions were seeking employees with a bachelor's degree (89%) with the remainder seeking an advanced (masters) degree (7%) or high school diploma (4%). Top skills required were fitness and wellness assessment, exercise programming and instruction, health coaching, and customer service. Examples of key responsibility areas included:

*“Provides tools, resources, and environments that support safe, efficient, healthy behaviors and encourage employees to proactively manage their health and wellbeing”.*

*“Will lead group exercise classes for employees, patients, and community members. Will educate participants on proper form, anatomy, contraindications, and modification of exercise moves”.*

*“Develop, coordinate, and evaluate programs focused on the prevention of work-related injuries, physical conditioning, and employee education and wellness within the manufacturing environment”*

#### Athletic performance or rehabilitation

Athletic Performance or Rehabilitation employees (*n* = 26) were focused on enhancing sports performance, general injury prevention and implementing rehabilitation protocols after sports related injuries. Positions included strength and conditioning coaches, athletic trainers, and athletics administration. Ninety two percent (92%) of job posting required a bachelor's degree, while 8% required an advanced or doctoral degree. The majority of positions requiring only an undergraduate degree were strength and conditioning coaches or athletic trainers, while those that required an advanced or doctoral degree were athletic trainers or nutrition focused.

Top skills required were developing strength and conditioning programs, athlete assessment, therapeutic programming, and athlete management. Examples of key responsibility areas included:

*“Performing assessments and programs that involve injury prevention, post rehab recovery and management of high performance athletes”*

*“Improve the overall development and performance for the athletes at all levels of competition”*

*“Will be observing and developing knowledge covering therapeutic assessments and treatments for a wide-ranging sports/orthopedic pathologies”.*

#### Clinical exercise specialist

Clinical exercise specialists (*n* = 108) were tasked with overseeing general exercise and rehabilitation programming for patients diagnosed with a disease or disorder. Positions were wide ranging and included cardiovascular/rehabilitation technicians, clinical exercise physiologists and physical therapy aids. Seventy four percent required a bachelor's degree while 22% required only a high school diploma and 4% required an advanced degree. Top skills required included medical terminology literacy and documentation ability, collaboration with healthcare professionals, completing non-invasive cardiac stress testing, designing exercise programs for individuals with clinical diagnoses. Examples of key responsibility areas included:

*“Assess and create exercise prescriptions for participants with medical complexity and/or physical disability”*

“*Provide direct patient care in the cardiac rehab environment and input to the interdisciplinary team regarding patient progress and ongoing care”*

*“Perform testing and interpretation of non-invasive cardiac exercise tests, musculoskeletal endurance, strength, flexibility, body composition, and analysis assessment”*

## Discussion

This study examined the updated the qualification requirements for professionals seeking employment in exercise science, exercise physiology, kinesiology or equivalent fields. It provides valuable insights into the evolving job market for exercise physiologists and exercise professionals in related fields, particularly as calls are being make globally to integrate the exercise professional into all aspects of healthcare (1–3, 12). The findings highlight the significant growth and transformation occurring in the field of exercise practice, driven by advancements in clinical research, increased awareness of physical fitness in overall health, and the rising demand for exercise-related interventions and programming. The projected 9% growth rate and the estimated 1,700 new job openings each year by 2031, as indicated by the Bureau of Labor Statistics (BLS), underscore the promising career prospects for individuals with an exercise degree (4). Further, it highlights areas of focus for university accreditation and training programs to best prepare students to meet the employers needs and demonstrates an opportunity to better align employer expectations with university training requirements.

The analysis of 615 job postings revealed several important trends and patterns. Firstly, there is a clear emphasis on the value of a bachelor's degree, with over 70% of the analyzed positions requiring this level of education. This finding represents a 24% increase compared to a decade ago (13), underscoring the increasing recognition of the importance of formal education in the field. This is an important shift in qualification standards for students graduating from programs in the US, and further aligns graduates with requirements seen abroad, where a master's degree is often a minimal qualification (14). Second, while entry-level certifications may not necessitate a college degree as a prerequisite, higher-level certifications often require one, in accordance with the new accreditation standards (6). Yet, even for jobs requiring a bachelor's or master's degree, many only then required an entry level certification (i.e., Certified Personal Trainer). This mismatch in degrees and certifications is similar to a recent report from the United Kingdom where staff roles and qualifications across services in the UK were found to be inconsistent (15). Addressing this misalignment will contribute to greater clarity and consistency in the industry, both in the US and abroad, particularly as degrees and certifications are becoming increasingly recognized across international borders (16).

Although four focus areas were identified: academic teaching and research, general fitness and worksite wellness, athletic performance and rehabilitation, clinical exercise specialist, the study also identified inconsistencies and overlaps in job titles as well as degree and certification requirements. Interestingly, clinical exercise specialists required the least amount of education, with 22% of positions posted requiring only a college degree. These practices can create confusion for both job seekers and employers and is a challenge for the field as the U.S. Bureau of Labor Statistics (4) provides the qualifications of a clinical exercise physiologist, including at least a bachelor's degree. Moreover in order to sit for a clinical exercise physiology certification, a bachelor's degree from an accredited university will be required (6, 7). Finally, despite the focus areas and certification requirements, employers also used several key terms interchangeably to advertise to the exercise professional including: exercise science, kinesiology, exercise physiology, exercise specialist, and exercise prescription. As the exercise field continues to evolve, there is a need for an established and universal occupational title and degree requirements for credentialed exercise professionals, particularly in the clinical space.

Specific certification requirements also varied greatly among the job criteria. For example, certifications related to exercise knowledge, such as certified personal trainer, exercise physiologist and certified strength and conditioning specialist, were sought after by employers despite the different educational requirements for each certification (i.e., no college degree, and a minimum of a bachelor's degree, respectively) and the job task analysis. Findings also identified several preferred certification issuers, including ACSM, NSCA, AFAA, ACE, NASM, and NETA, notwithstanding the varying levels of credentialing and academic rigor required by each. In addition to being confusing to professionals seeking employment, the lack of consistent standards and requirements makes it difficult for universities to attract students to specific programs of study and adequately create exercise science curricula to best prepare graduates for the job market. Further, the differing credentialing expectations of employers potentially dilutes the value of the certifications because their perceived value in the labor market remains unclear (17).

While this study provides valuable insights, certain limitations should be acknowledged. The research captured a snapshot in time, specifically in the post-pandemic period when the fitness industry experienced significant disruption. Additionally, the analysis did not encompass all possible search engines, which may have influenced the results. Future research could address these limitations by conducting longitudinal studies and expanding the scope of search engines to provide a more comprehensive understanding of the job market.

To facilitate a smoother transition for students, universities, job seekers and employers, it is crucial to increase outreach efforts aimed at informing employers about the specific credentialing and accreditation requirements in the field. Organizations such as the Coalition for the Registration of Exercise Professionals® (CREP®) (18) and recent developments such as the Health Level Seven International (HL7) global network of healthcare databases formally approving a Physical Activity Implementation Guide (19) can play a pivotal role in disseminating this information and promoting better alignment between educational qualifications and certification standards. By addressing these issues, stakeholders can actively contribute to a more coherent and well-informed exercise science job market, fostering better opportunities for professionals and promoting the continued growth and development of the exercise field. Finally, an alignment of exercise specific job titles, expectations, and qualifications, both in the US and abroad, can inform best service practices that ultimately result in the improved the health of clients and patients.

## Data Availability

The raw data supporting the conclusions of this article will be made available by the authors, without undue reservation.
